# Circulating Plasma Proteins as Biomarkers for Immunotherapy Toxicity: Insights from Proteome-Wide Mendelian Randomization and Bioinformatics Analysis

**DOI:** 10.3390/biomedicines13071717

**Published:** 2025-07-14

**Authors:** Liansha Tang, Wenbo He, Handan Hu, Jiyan Liu, Zhike Li

**Affiliations:** 1 Department of Biotherapy, Cancer Center, West China Hospital of Sichuan University, Chengdu 610041, China; tangliansha@stu.scu.edu.cn (L.T.); huhandanaz@163.com (H.H.); 2 Department of Neurosurgery, West China Hospital of Sichuan University, Chengdu 610041, China; hewenboscu@163.com; 3 Department of Oncology, The First Affiliated Hospital of North Sichuan Medical College, Nanchong 637000, China

**Keywords:** plasma protein, inflammatory cytokine, immune-related adverse events, mendelian, colocalization

## Abstract

**Background:** Immune checkpoint inhibitors (ICIs) have transformed cancer treatment, yet severe immune-related adverse events (irAEs) often necessitate immunotherapy discontinuation and cause life-threatening complications. Circulating plasma proteins, dynamically accessible and functionally linked to immunity, may predict and offer novel targets for irAEs. **Methods:** Leveraging multi-omics integration, we conducted bidirectional two-sample Mendelian randomization (MR) using protein quantitative trait loci (pQTLs) from 4998 plasma proteins and genome-wide association data of irAE phenotypes. A causal inference framework combining colocalization analysis, multivariable MR (MVMR) adjusting for body mass index (BMI) confounding, and mediation MR elucidated BMI-independent pathways. Systems biology approaches including tissue-specific expression profiling, pathway enrichment, and protein interaction network analysis revealed spatial and functional drivers of irAE pathogenesis. **Results:** Proteome-wide MR mapping identified eight plasma proteins (CCL20, CSF1, CXCL9, CD40, TGFβ1, CLSTN2, TNFSF12, TGFα) causally associated with all-grade irAEs, and five (CCL20, CCL25, CXCL10, ADA, TGFα) with high-grade irAEs. Colocalization prioritized CD40/TNFSF12 (all-grade) and ADA/CCL25 (high-grade) as therapeutic targets (PPH4 > 0.7). CXCL9/TNFSF12 (all-grade) and CCL25 (high-grade) exerted BMI-independent effects, suggesting intrinsic immune dysregulation mechanisms. Tissue-specific gene expression patterns, CSF1, TGFβ1 in lung, TNFSF12 in the ileum may explain organ-specific irAE vulnerabilities. High-grade irAEs correlated with compartmentalized immune dysregulation and IL-17/immunodeficiency pathway activation. **Conclusions:** This study establishes the causal atlas of plasma proteins in irAE pathogenesis, bridging biomarker discovery with actionable therapeutic targets. These advances align with next-generation immunotherapy goals: maximizing efficacy while taming the immune storm.

## 1. Introduction

Recent advances in immune checkpoint inhibitors (ICIs) have revolutionized cancer treatment, significantly improving survival outcomes in patients with advanced malignancies. However, ICI therapy can lead to the breakdown of immune tolerance, triggering immune-mediated responses against non-tumor cells, known as immune-related adverse events (irAEs) [1]. These adverse events can affect any organ. Studies indicated that approximately 54–76% of ICI-treated patients experienced varying degrees of toxicity [2], with 10–30% classified as severe (grade ≥3) [3], potentially leading to long-term immune complications or even death [4]. Although predictive models based on clinical features, such as tumor type and PD–L1 expression, have progressed in risk stratification [5], their accuracy is often limited by individual variability and the complexity of dynamic immune interactions. Consequently, identifying biomarkers capable of early detection, precise stratification, and guiding interventions has become a crucial scientific goal in optimizing the safety of immunotherapy.

Circulating plasma proteins, serving as “dynamic molecular sensors” of immune status and pathological processes, offer distinct advantages in predicting and elucidating the mechanisms of irAEs. Elevated levels of IL–6 and CXCL5 following ICI treatment were identified as early indicators of impending irAEs [6,7]. In patients with immune-related diarrhea/colitis, a decline in IL–17 levels was a marker of symptom resolution [8]. In addition, CRP also exhibited significant abnormalities before clinical symptoms appeared [9]. In melanoma cohorts, 42% of patients showed elevated CRP levels prior to the onset of clinical manifestations. Furthermore, IFN–γ was considered a protective factor against ICI-induced pneumonia, with lower levels of IFN-γ (<10 U/mL) associated with an increased risk of pneumonia [10]. However, traditional studies face several challenges: first, serum proteomics are highly sensitive to sample handling, making cross-cohort analyses costly and prone to technical biases; second, protein expression differences in case-control studies may be confounded by reverse causality related to irAEs pathology; and third, comorbidities, concomitant medications, and other confounding factors significantly limit the ability to draw causal inferences in observational research.

Mendelian randomization (MR) provides an innovative solution to address these challenges. Its underlying principle, based on the random allocation of alleles during gamete formation [11], allows for causal inference between exposures (plasma proteins) and outcomes (irAEs), mimicking the strength of a randomized controlled trial. This approach effectively mitigates confounding bias and reverse causality issues commonly encountered in traditional studies. Notably, existing GWAS evidence has identified significant genetic variations in immune-regulatory gene regions (IL7 and IL22RA1), with carriers of these variants exhibiting a nearly twofold increased risk of developing irAEs (HR = 1.8–2.1) [12,13]. Furthermore, recent large-scale proteomics studies have uncovered thousands of quantitative trait loci associated with circulating protein levels (pQTL) [14,15,16], many of which have been shown to have causal links with autoimmune diseases, cardiovascular disorders, and cancer progression in multiple MR studies [17,18,19]. The broad applicability of this “protein-disease” causal network forms the theoretical foundation for this study.

We integrated large-scale pQTL data with publicly available irAEs GWAS datasets to systematically identify plasma protein biomarkers significantly associated with irAEs. Using two-sample MR, colocalization, and pathway enrichment analyses, this study aimed to uncover the underlying immune pathways, such as cytokine storms. These findings will provide valuable scientific evidence to inform the development of personalized monitoring strategies and optimize the risk-benefit balance of immunotherapy.

## 2. Materials and Methods

### 2.1. GWAS Identification for Circulating Proteins and irAEs

#### 2.1.1. Circulating Proteins

Our study was conducted in accordance with the MR-STROBE guidelines. Genetic instruments for circulating proteins, derived from protein quantitative trait loci (pQTLs), were obtained from two independent proteome-wide association studies [15,16]. A total of 4907 plasma proteins were provided by Ferkingstad et al. [15] from 35,559 Icelandic participants. The SomaLogic platform measured protein concentrations in plasma samples using 5284 aptamers, with the results expressed as relative fluorescence units quantified on a DNA microarray. Quality control was performed by calculating the correlation of aptamers between samples, excluding those with low correlation (below 0.82) and multi-gene mapping aptamers. Reproducibility testing of 228 duplicate samples confirmed the good performance of the SomaLogic platform. Furthermore, measurements from the SomaLogic and Olink Target platforms were compared, confirming their similarity. This study employed Illumina technology for genotyping, with joint genotyping and long-range phasing used to improve accuracy. Genotype imputation was performed to supplement the genotypes of unsequenced individuals and rare variant information, ensuring that only high-confidence variants (MAF > 0.01% and imputation quality > 0.9) were used.

In addition, we also included 91 circulating inflammatory proteins reported by Zhao et al. [16], which were measured utilizing the Olink Target Inflammation platform in 14,824 European participants from 11 cohorts. In this study, quality control was implemented by excluding proteins below the Lower Limit of Detection (LLOD) and normalizing all data to relative units on a log scale, thereby improving data comparability. Reproducibility was assessed by comparing the pQTL results with those from other studies. Genotyping was performed using SNP arrays for each cohort, and imputation methods similar to those used in previous studies were applied. Both GWAS studies on circulating plasma proteins were adjusted for age and sex to improve the accuracy and reliability of the results.

#### 2.1.2. irAEs

Our study leveraged summary-level data on immune-related adverse events (irAEs) from a genome-wide association analysis (GWAS) conducted at the Dana-Farber Cancer Institute (DFCI) [12]. The cohort consisted of 1751 individuals of European ancestry diagnosed with cancer and treated with ICIs from 2013 to 2020. Within this cohort, a PD–1/PD–L1 inhibitor was administered to approximately 90% of patients, whereas the remaining 10% received dual therapy combining PD–1/PD–L1 and CTLA–4 inhibitors. Using an algorithm-based analysis of electronic health records, 339 patients were identified as having experienced irAEs of any grade, with the majority being grade 2 or higher. According to the National Cancer Institute’s Common Terminology Criteria for Adverse Events (CTCAE v.5), 259 patients developed severe irAEs (grade ≥ 3), and all cases were meticulously documented. Furthermore, tumor tissue samples from all patients were subjected to targeted sequencing using the OncoPanel platform. After rigorous quality control, germline single-nucleotide polymorphisms (SNPs) were inferred from ultra-low-coverage off-target reads. A GWAS was subsequently performed in the DFCI cohort to investigate the association between genetic variants and the time from ICI initiation to the onset of irAEs.

### 2.2. Instrumental Variables Selection

To ensure dataset consistency, all SNPs were mapped to the human genome Build 37 (NCBI GRCh37) as a unified reference. This alignment standardized genotype data across GWAS sources, eliminating potential biases from reference genome version differences. The IVs were selected according to the following criteria: (1) To ensure an adequate number of SNPs for robust analysis, the significance threshold for IVs was set at *p* < 5 × 10^−6^ [20]. (2) To account for linkage disequilibrium (LD), SNPs with an r^2^ < 0.001 within a 10,000 kb window were retained, ensuring genetic independence between loci and minimizing correlations in allele frequencies. (3) The strength of the IVs was assessed using F-statistics, where higher values indicate stronger predictive power. Only IVs with an F-statistic > 10 were retained to mitigate weak instrument bias and ensure the reliability of the Mendelian randomization (MR) results [21,22]. (4) SNPs were selected based on their association with the exposure, and those exhibiting a *p*-value <5 × 10^−8^ for the outcome were excluded to prevent direct effects on the outcome. This step ensures that the selected SNPs influence the outcome exclusively through exposure, preserving the core assumptions of MR. (5) To address potential confounding, SNPs associated with known confounders were identified and removed using PhenoScanner V2.

To eliminate technical biases during integration of SomaLogic and Olink pQTL datasets, inverse-rank normal transformation was applied for cross-platform batch effect correction, with effect estimates subsequently scaled to standard deviation units. During data integration, we used GRCh37 as a unified reference genome to align genotype coordinates across GWAS sources, eliminating potential biases from reference version differences. Simultaneously, TwoSample MR implemented strand orientation and allele frequency adjustments.

### 2.3. MR Analysis

#### 2.3.1. Two-Sample MR Analysis

We utilized the R package “TwoSampleMR” (version 0.6.8) to conduct MR analysis, aiming to assess the relationships between genetically predicted plasma protein levels and irAEs. We applied multiple approaches, including inverse-variance-weighted (IVW), MR Egger, weighted median, simple mode, and weighted mode, to infer causality. Among these, IVW was considered the primary method [23]. The odds ratios (OR s) for the elevated risk of irAEs were calculated based on a one standard deviation (SD) increase in circulating protein levels. In addition, we applied the Benjamini–Hochberg procedure to correct for multiple comparisons using the false discovery rate (FDR), with *p*-values < 0.05 considered statistically significant for causality.

#### 2.3.2. Multivariable and Mediation Mendelian Randomization

In multivariable Mendelian randomization (MVMR), multiple genetic instruments can be employed, even if they are not associated with the same exposure [24]. However, all IVs must satisfy the core MR assumptions to ensure valid causal inference, including relevance, exclusion restriction, and independence from confounders [25]. Given that the plasma protein exposure GWAS has already adjusted for age and sex, we additionally adjusted for BMI in the MVMR analysis to determine their independent effects on irAEs [26]. MVMR analysis utilized SNPs associated with both BMI and at least one plasma protein, which were then combined as IVs for each exposure. Two-step MR was performed to identify the mediating effect of BMI in the significant protein-irAEs. The total effect of proteins on the irAEs was estimated using univariable MR. Two-sample MR was performed for plasma proteins and BMI, as well as for BMI and irAEs, to estimate the indirect effect, which was calculated by multiplying the two-step MR estimates [27]. Stepwise regression was applied to identify the exposures and mediators with significant effects. The direct effect was then derived by subtracting the indirect effect from the total effect.

### 2.4. Sensitivity Analysis

A sensitivity analysis was performed to assess heterogeneity and pleiotropy. Cochran’s Q test with random-effects IVW was used to detect heterogeneity (*p* < 0.05). MR-PRESSO identified outliers and addressed horizontal pleiotropy, while MR-Egger’s intercept assessed vertical pleiotropy. Leave-one-out analysis examined the influence of individual SNPs, and scatter and funnel plots were used to evaluate result consistency and homogeneity. Reverse causality between circulating plasma proteins and irAEs was examined using the same analytical method.

### 2.5. Colocalization Analysis

We performed colocalization analysis using the coloc R package to assess the association between genomic loci and traits (such as protein levels, irAEs, or high-grade irAEs) and determine whether shared causal variants drive these associations [28]. This approach helps confirm causal relationships rather than associations due to linkage disequilibrium (LD) or confounding factors. We analyzed pQTL SNPs within 1 MB regions, with minor allele frequencies (MAF) > 0.01 [29]. The method evaluates five hypotheses: H0: no association; H1: associated with irAEs or high-grade irAEs only; H2: associated with protein levels only; H3: associated with both traits but with distinct causal variants; H4: associated with both traits and sharing the same causal variant. Posterior probabilities (PP) were calculated for each hypothesis. PP.H4 ≥0.7 indicates strong evidence for shared causal variants, 0.5< PP.H4 <0.7 indicates moderate evidence, and PP.H4 <0.5 suggests weak evidence [30]. Proteins were classified based on the strength of colocalization evidence into level 1 (strong), level 2 (moderate), and level 3 (weak) targets.

### 2.6. Pathway and Interaction Analysis

#### 2.6.1. Pathway and Enrichment Analysis

We employed the KEGG Orthology-Based Annotation System (KOBAS) to conduct Gene Ontology (GO) and Kyoto Encyclopedia of Genes and Genomes (KEGG) enrichment analyses, with the goal of gaining deeper insights into the biological functions and metabolic pathways of proteins exhibiting similar expression profiles [31]. GO analysis was utilized to examine the shared characteristics of genes across three main categories: biological processes (BPs), molecular functions (MFs), and cellular components (CCs). This analysis assessed gene enrichment within each GO category by comparing the target genes to the reference genome, generating relevant enrichment results. KEGG enrichment analysis, on the other hand, focused on identifying metabolic pathways in which the genes were significantly enriched [32]. Enrichment analyses were performed on proteins that showed a positive correlation with immune-related adverse events (irAEs), and only those results with a corrected *p*-value <0.05 were retained for further interpretation.

#### 2.6.2. Clustered Tissue Expression Analysis

We conducted enrichment analysis using FUMA8 (https://fuma.ctglab.nl), prioritizing genes within biological pathways and functional categories based on gene sets from MSIGDB21 and WikiPathways [33]. To assess whether the pQTLs of irAEs-prioritized proteins serve as expression quantitative trait loci (eQTLs), we integrated Genotype-Tissue Expression (GTEx) V8 data in FUMA8 to construct gene expression heatmaps [34]. Since pQTLs and eQTLs are typically regulated by the same genetic variants influencing protein and mRNA expression, the GTEx data allowed us to compare changes in gene expression across different tissues and their corresponding protein levels, providing evidence for the potential eQTL role of the pQTLs.

#### 2.6.3. Gene Network Analysis

The Gene Multiple Association Network Integration Algorithm (GeneMANIA) tool (http://www.genemania.org/) was utilized to construct a gene network for investigating the prioritized protein functions and protein–protein interactions in irAEs from both MR and colocalization [35,36]. This analysis identifies a group of genes/proteins that are potentially functionally related to the selected ones, based on their interaction profiles. It assigns continuous values ranging from 0 to 1 to reflect the degree of co-regulation between these genes.

### 2.7. Construction of the Causal Risk Score

We built a causal risk score (CRS) based on MR results. The core formula is: CRS = Σ [w_i × Protein_i]. Protein_i represents the standardized concentration of the i-th plasma protein, and w_i is the causal effect estimate (β_IVW) for irAEs derived via IVW MR. This weight reflects the protein’s causal contribution per SD increase.

## 3. Results

### 3.1. Identification of Instrumental Variables

**Figure 1:**
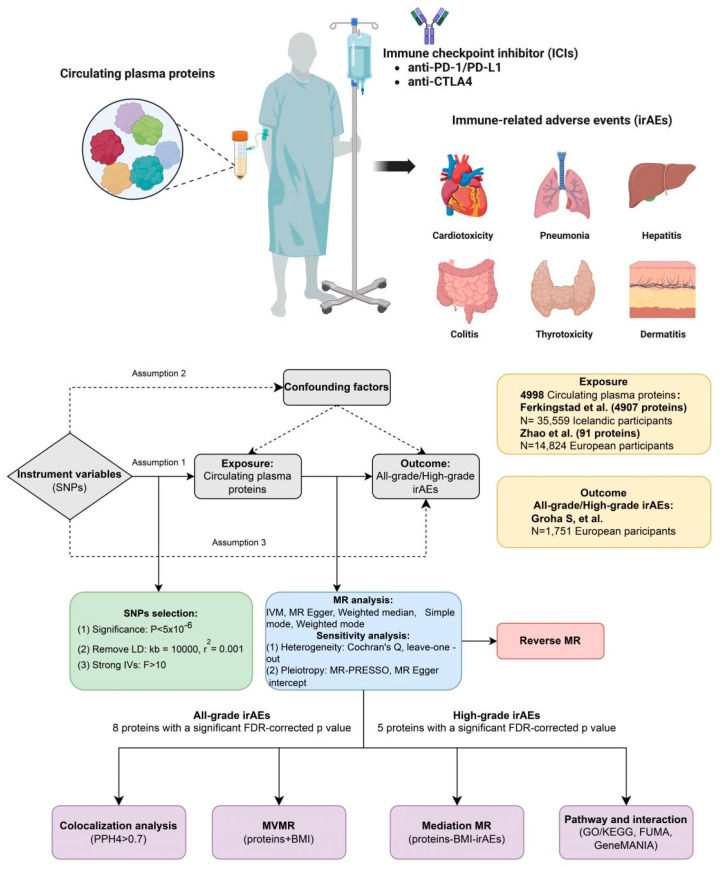
Overview of MR design.

The study flow chart was presented in Figure 1. Through comprehensive screening, we identified a set of instrumental variables (IVs) associated with exposures (Table S1). A total of 17,948 SNPs associated with 3482 circulating plasma proteins were included in our analysis. The minimum value of F statistics from these SNPs was 20, well exceeding the standard threshold of 10, which indicated no significant instrument bias.

### 3.2. Causal Effects of Circulating Proteins on All-Grade irAEs

**Figure 2:**
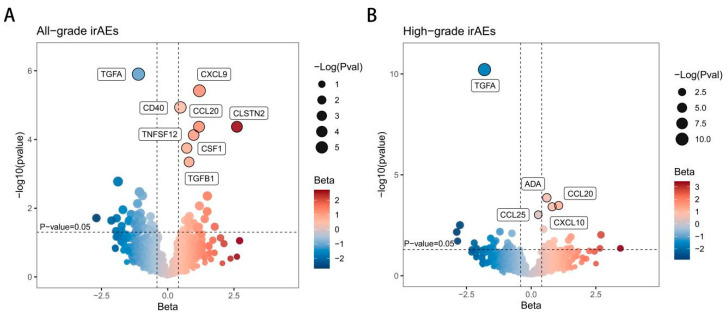
The volcano plot of the MR results. (**A**) Circulating plasma proteins and all-grade irAEs. (**B**) Circulating plasma proteins and high-grade irAEs. The horizontal coordinate is the Beta value of the MR Result, and the vertical coordinate is -log10 (*p* value). Beta values less than 0 are shown in blue (protective factors), and beta values greater than 0 are shown in red (risk factors). The larger the absolute value of Beta, the darker the color. Positive protein indicators are highlighted with boxes.

**Figure 3:**
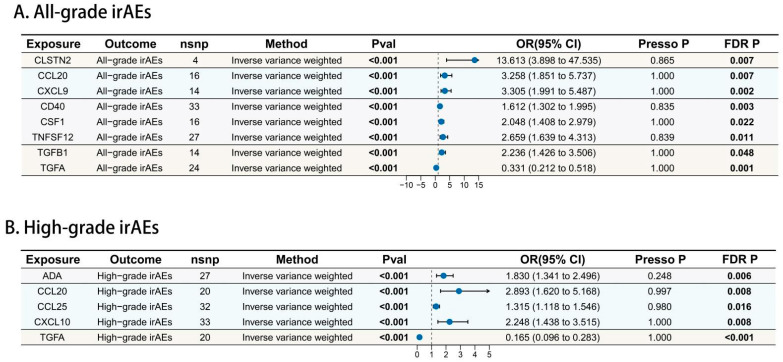
The forest plot showed the positive results of MR analysis. (**A**) All-grade irAEs. (**B**) High-grade irAEs. The background colors of different proteins represent different protein classifications, light yellow (CLSTN2) represents new protein indicators, and light blue (CCL20, CCL25, CXCL9, CXCL10) represents chemokines. Light purple represents immune receptor-ligand (CD40, CSF1, TNFSF12, ADA), light green represents growth factors (TGFβ1, TGFα).

The causal effects of plasma proteins on all-grade irAEs were shown in Figure 2A. The SNPs used in the MR analysis are in Table S2. Using IVW as the primary MR method, the effect estimates across all five MR approaches were aligned in direction. The results showed eight circulating plasma proteins were positively associated with the risk of all-grade irAEs (Figure 3A and Table S3). Chemokines, including CCL20 (OR = 3.258,95% CI: 1.851–5.737, *p* = 4.26 × 10^−5^), CSF1 (OR = 2.048, 95% CI: 1.408–2.979, *p* = 1.78 × 10^−4^), and CXCL9 (OR = 3.305, 95% CI: 1.991–5.487, *p* = 3.81×10^−6^), the immune receptor CD40 (OR = 1.612, 95% CI: 1.302–1.995, *p* = 1.16 × 10^−5^), the growth factor TGFβ1 (OR = 2.236, 95% CI: 1.426–3.506, *p* = 4.51 × 10^−4^), the neuro-related protein CLSTN2 (OR = 13.613, 95% CI: 3.898–47.535, *p* =4.27 × 10^−5^), and the inflammation-regulating factor TNFSF12 (OR = 2.659, 95% CI: 1.639–4.313, *p* = 7.43 × 10^−5^) are identified as risk factors for all-grade irAEs. The growth factor TGFα (OR = 0.331, 95% CI: 0.212–0.518, *p* = 1.25 × 10^−6^) was the protective factor for all-grade irAEs. After FDR correction, the *p*-value was still significant (<0.05), indicating the robustness of our results. The sensitivity analysis showed no significant heterogeneity and pleiotropy bias (*p* > 0.05) (Tables S4 and S5).

### 3.3. Causal Effects of Circulating Proteins on High-Grade irAEs

Figure 2B presents the causal relationship between plasma proteins and high-grade irAEs. Table S6 presents the SNPs utilized in this MR analysis. Five circulating proteins showed a significant causal relationship with high-grade irAEs (Figure 3B and Table S7). Chemokines, including CCL20 (OR = 2.893, 95% CI: 1.620–5.168, *p* = 3.32 × 10^−4^), CCL25 (OR = 1.315, 95% CI: 1.118–1.546, *p* = 9.49 × 10^−4^), and CXCL10 (OR = 2.248, 95% CI: 1.438–3.515, *p* = 3.81 × 10^−4^), as well as adenosine metabolism-associated factor ADA (OR = 1.830, 95% CI: 1.341–2.496, *p* = 1.36 × 10^−4^) could increase the risk of high-grade irAEs. Elevated growth factor TGFα level (OR = 0.165, 95% CI: 0.096–0.283, *p* = 9.12 × 10^−11^) still indicated the decreased risk of high-grade irAEs. No significant heterogeneity and pleiotropy bias were found in the sensitivity analysis (Tables S8 and S9).

### 3.4. Reverse MR Analysis

Reverse MR analysis was conducted to examine the causal relationship between circulating proteins and all-grade irAEs, as well as high-grade irAEs (Tables S10 and S11). We did not find a positive relationship between plasma proteins and irAEs.

### 3.5. Colocalization Analysis

Colocalization analysis was performed for all-grade irAEs and high-grade irAEs with circulating proteins (Table 1 and Table 2). The colocalization evidence was found in two proteins for all-irAEs: CD40 (pQTL: rs1883832, PP.H4 = 0.716) and TNFSF12 (pQTL: rs62061198, PP.H4 = 0.859). ADA (pQTL: rs11555566, PP.H4 = 0.817) and CCL25 (pQTL: rs2032887, PP.H4 = 0.739) were identified as the leading candidates for high-grade irAEs.

### 3.6. Multivariable and Mediation MR Analysis

To control for confounding factors, we performed an MVMR analysis, including both BMI and the proteins (Tables S12 and S13). The results showed that two proteins, CXCL9 (OR = 1.723, 95% CI: 1.265–3.032, *p* = 0.001) and TNFSF12 (OR = 1.663, 95% CI: 1.132–2.524, *p* = 0.004), maintained a significant effect on all-grade irAEs after adjusting for BMI. CCL25 (OR =1.106, 95% CI: 1.009–1.181, *p* = 0.044) kept a significant causal relationship with high-grade irAEs. Furthermore, a two-step mediation MR analysis was conducted to identify the mediating effect of BMI on protein–irAEs associations (Tables S14 and S15). However, no significant mediating effects were found between the proteins and all-grade irAEs, or between the proteins and high-grade irAEs.

### 3.7. Pathway and Interaction Analysis

**Figure 4:**
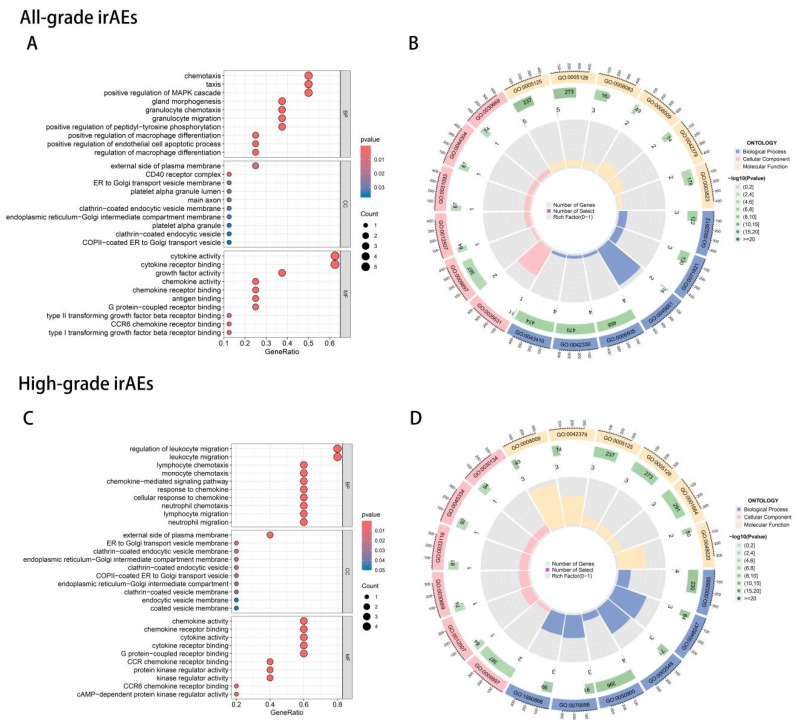
The GO enrichment analysis of 8 identified proteins for all-grade irAEs (**A**) and 5 identified proteins for high-grade irAEs (**C**). Significantly enriched pathways were shown in red. (**B**,**D**) are cyclic graphs corresponding to the enrichment of all-grade irAEs and high-grade irAEs. The outermost ring indicated the GO terms of enrichment. The innermost circle represented the degree of three GO enrichment categories, with blue representing biological process, pink representing cellular component, and yellow representing molecular function. The higher the column, the higher the degree of enrichment in this category. The number in the green background of the inner circle indicated the number of genes enriched under this term, and the shade of green represented the size of the *p*-value, and the darker the color, the smaller the *p*-value. The next circle represented the number of protein genes enriched in this pathway. The larger the number, the wider the pink in the background.

For all-grade irAEs (Figure 4A,B), biological processes such as chemotaxis and granulocyte chemotaxis were enriched, highlighting immune cell migration. Membrane-associated components, including the plasma membrane and CD40 receptor complex, were also enriched, emphasizing immune receptor interactions. In high-grade irAEs (Figure 4C,D), leukocyte migration, particularly lymphocyte and monocyte chemotaxis, was more strongly enriched, indicating a more specific immune response. Endocytic vesicle components were notably enriched, suggesting differences in protein trafficking. The main distinction was the stronger focus on leukocyte migration and endocytic vesicle components in high-grade irAEs, indicating a more complex immune response.

**Figure 5:**
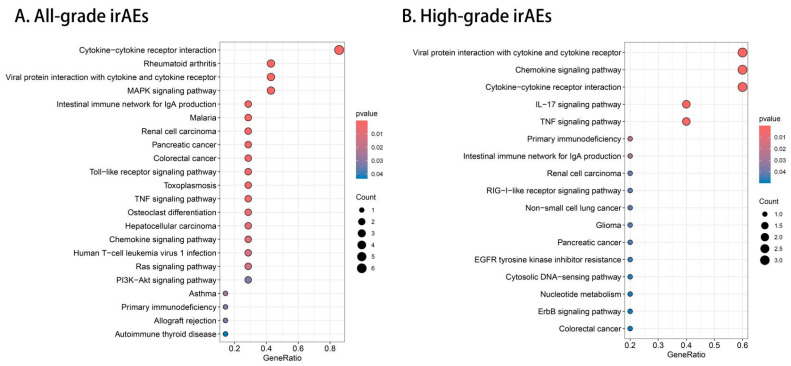
The KEGG enrichment analysis of 8 identified proteins for all-grade irAEs (**A**) and 5 identified proteins for high-grade irAEs (**B**). Significantly enriched pathways were shown in red.

The KEGG pathway analysis of all-grade irAEs (Figure 5A) and high-grade irAEs (Figure 5B) demonstrated that the cytokine–cytokine receptor interaction pathway was central to both, but high-grade irAEs showed significant enrichment in the viral protein interaction with cytokine receptors and IL–17 signaling pathways, the latter of which was not enriched in irAEs. Additionally, high-grade irAEs exhibited enrichment in pathways related to primary immunodeficiency and immune system dysfunction, indicating that high-grade irAEs were associated with more specific immune dysregulation mechanisms.

**Figure 6:**
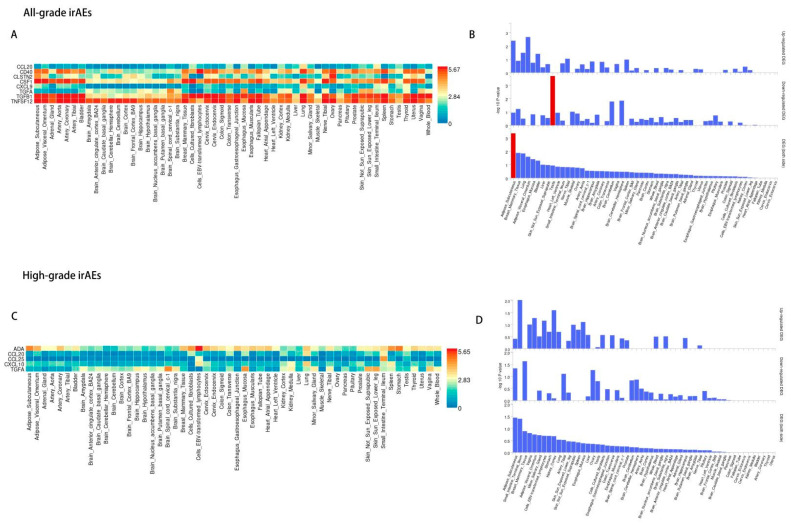
Clustered tissue expression heatmaps of the pQTL of the candidate proteins for all-grade irAEs and high-grade irAEs from FUMA. The graph (**A**) for all-grade irAEs and (**C**) for high-grade irAEs) depicted normalized expression values (zero-mean normalization of log2-transformed expression), where the darker red signified higher relative expression of the gene in each label, compared to a darker blue color in the same label. The graph (**B**) for all-grade irAEs and (**D**) for high-grade irAEs) depicted -log10 *p*-values of Differentially Expressed Gene (DEG) sets for each expression dataset. “Up-regulated” denoted over-expression and “Down-regulated” denoted under-expression.

Utilizing FUMA, the genes of all-grade irAEs-associated proteins, especially CSF1, TGFβ1, TNFSF12, and CD40, were predominantly expressed in the following tissues: adipose subcutaneous tissue, breast mammary tissue, and lung tissue (Figure 6A,B and Table S16). In terms of high-grade irAEs (Figure 6C,D and Table S17), the genes with higher expression were observed in tissues like adipose subcutaneous tissue, small intestine terminal ileum, and breast mammary tissue.

**Figure 7:**
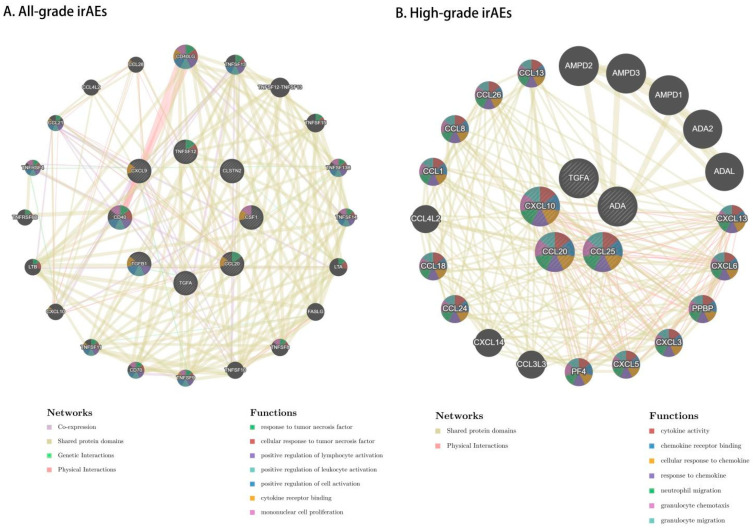
Protein–protein interactions and gene network analyses using GeneMANIA for 8 and 5 candidate proteins for all-grade irAEs (**A**) and high-grade irAEs (**B**), respectively. The genes of the MR-prioritized proteins are shown as larger inner circles, while genes from the GeneMANIA extension are smaller and appear in the outer circle. Here, the red line represents the physical interaction, the purple line represents co-expression, the green line represents the genetic interaction, and the yellow line represents shared protein domains. The background colors of each protein correspond to different functions.

GeneMANIA showed that most of the genes involved in the eight candidate proteins related to all-grade irAEs had co-expression, shared protein domains, and genetic and physical interaction (Figure 7A and Table S18). The functions of these proteins included the positive regulation of immune cell activation and interaction. Five candidate proteins associated with high-grade irAEs had similar protein networks with all-grade irAEs (Figure 7B and Table S19). The protein functions focused on the cytokine activity and chemokine regulation.

### 3.8. Causal Weights for Risk Prediction

We computed causal weights (w_i) for all significant plasma proteins (IVW-MR FDR < 0.05) to enable future CRS construction. These parameters, cataloged in Table S20, represent each protein’s contribution to irAEs risk per SD increase. Notably, clinical validation of the CRS requires individual-level protein and outcome data, which are currently unavailable. Researchers can directly apply the provided weights and CRS formula described herein to generate patient-specific risk scores and comprehensively evaluate predictive performance.

## 4. Discussion

Our MR study utilized pQTL as IVs to explore the putative causal relationship of circulating plasma proteins on all-grade irAEs and high-grade irAEs. The results provided evidence for the association between eight plasma proteins (CCL20, CSF1, CXCL9, CD40, TGFβ1, CLSTN2, TNFSF12, and TGFα) and all-grade irAEs, and between five plasma proteins (CCL20, CCL25, CXCL10, ADA, and TGFα) and high-grade irAEs. Crucially, colocalization confirmed that genetic variants driving protein levels of CD40, TNFSF12, ADA, and CCL25 directly influence irAE risk, with ADA and CCL25 specifically linked to high-grade irAEs. MVMR further revealed that CXCL9, TNFSF12 (all-grade), and CCL25 (high-grade) exert BMI-independent effects, implicating direct immunomodulatory pathways rather than obesity-related inflammation. Pathway analyses further delineated severity-specific mechanisms, with high-grade irAEs uniquely enriched for IL-17 signaling and primary immunodeficiency pathways.

### 4.1. Chemokine

Integrating MVMR, colocalization, and pathway analyses, CXCL9 and CCL25 were prioritized as central chemokines orchestrating irAE pathogenesis. Similarly, several studies [6,37] identified that CXCL9/CXCL10 experienced an early increase, nearly 2 weeks after the start of immunotherapy, suggesting these chemokines were early response molecules involved in the immune system activation by ICIs. ICIs enhance antitumor immunity by disinhibiting T cells, leading to increased IFN-γ secretion. IFN-γ subsequently stimulates dendritic cells (DCs) and macrophages to produce CXCL9/CXCL10, which, combined with its receptor CXCR3, recruits Th1 cells, CD8+ T cells, and NK cells into normal tissues [38]. This infiltrate drives inflammatory damage through direct cytotoxicity (e.g., granzyme/perforin release) and secondary cytokine storms (e.g., TNF-α, IL-6) [39]. Crucially, infiltrating T cells further amplify IFN-γ production, establishing a self-reinforcing inflammatory loop that exacerbates tissue injury. Clinically, elevated CXCL9/CXCL10 levels have been detected in the serum of patients with ICI-induced arthritis [38] and in lesional skin biopsies from those with immunotherapy-related dermatitis [40], directly linking this chemokine to irAE pathogenesis across organs. Notably, patients with multi-organ irAEs presented a significant CXCL10 increase [6,37,41], suggesting that it could be a promising biomarker to predict high-grade irAEs, which is consistent with our results.

CCL25, primarily expressed in the small intestinal epithelium [42], regulates gut-specific T cell trafficking and maintains intestinal homeostasis via interactions with its receptors CCR9 and α4β7 integrin [43,44]. Our results showed the dual role of CCL25 in immunotherapy. The elevated level of CCL25 increased the risk of high-grade irAEs independent of BMI. This finding is consistent with Wurbel et al.’s observations in an acute colitis model, where elevated expression of CCL25/CCR9 during the inflammatory phase suggested that it may drive immune response dysregulation under pathological conditions [45]. Mechanistically, ICI therapy amplifies IFN-γ production, which induces CCL25 overexpression in intestinal epithelial and stromal cells. Elevated CCL25 recruits CCR9+ Th17 cells and cytotoxic T lymphocytes (CTLs) to the colonic mucosa, triggering IL-17/IFN-γ-mediated epithelial damage and macrophage activation. Critically, the dynamic rise of CCL25 post-ICI in serum may serve as an early biomarker to guide preemptive interventions, offering a window to mitigate toxicity without compromising antitumor efficacy.

Similar to CCL25, the CCL20/CCR6 axis is involved in providing homing signals of immune cells, including both suppressive and proinflammatory T cell subsets under inflammatory conditions [45,46,47]. CCL20 exacerbates tissue damage by suppressing Foxp3 expression in Treg cells, driving their differentiation into pro-inflammatory Th17-like Treg cells. Concurrently, CCL20 promotes the transition of Th17 cells into a highly pathogenic Th1-like Th17 subset, which secretes IL–17 and IFN–γ to drive epithelial barrier disruption and macrophage hyperactivation. This Treg/Th17 imbalance is strongly implicated in irAEs pathogenesis [48,49], particularly in colitis, where serum IL–17 levels correlate significantly with disease severity. Previous studies have demonstrated that CCL20 is markedly upregulated in autoimmune disorders such as inflammatory bowel disease (IBD) and arthritis, and anti-CCL20 antibodies have shown efficacy in ameliorating symptoms in clinical trials [45,47]. Given the shared Th17-driven inflammatory pathology between irAEs and autoimmune diseases, targeting CCL20 represents a possible therapeutic strategy for precision management.

### 4.2. Immune Receptor–Ligand Interactions

TNFSF12 (TWEAK), a member of the TNF superfamily, emerged as a significant risk factor for all-grade irAEs in our MR analysis. TNFSF12 is mainly produced by monocytes and macrophages [50]. Its receptor Fn14 is found in non-hematopoietic cells, including tissue progenitor cells, skeletal muscle cells, and endothelial cells. The TNFSF12–Fn14 interaction activates the NF-κB signaling pathway, driving the secretion of proinflammatory cytokines such as monocyte chemoattractant protein-1 (MCP–1/CCL2), IL-6, and TNF-α, while enhancing macrophage chemotaxis and polarization toward proinflammatory phenotypes (M1) [51]. This axis has been implicated in the pathogenesis of immune-associated myositis, IBD, and arthritis [52,53,54]. Our colocalization analysis further supports shared causal variants between TWEAK and all-grade irAEs, indicating that genetic predisposition to elevated TWEAK levels may directly contribute to irAE pathogenesis. Notably, the TNF-α blockade using agents like infliximab has become a recommended strategy for managing severe or refractory immune-related colitis and arthritis in patients receiving ICIs [55]. Emerging therapeutic approaches targeting the TWEAK/Fn14 axis show promise for irAEs. Preclinical and clinical studies have demonstrated that anti-TWEAK monoclonal antibodies effectively reduce tissue infiltration of pathogenic macrophages in rheumatoid arthritis [56] and autoimmune encephalomyelitis [57], exhibiting therapeutic potential in managing irAEs.

CD40, also a pivotal co-stimulatory molecule of the TNF receptor superfamily, is broadly expressed on immune cells (e.g., B cells, DCs) and non-immune cells (intestinal epithelial cells, synovial cells) [58]. Its interaction with the ligand CD40L induces IFN-γ secretion, matrix metalloproteinase (MMP) production, and chemokine release, promoting inflammatory cell infiltration and tissue damage. Our MR study demonstrated colocalization between CD40 genetic variants and all-grade irAEs (PP.H4 > 0.7), suggesting a causal relationship. Pathologically, CD40L overexpression correlates with increased disease activity in systemic lupus erythematosus (SLE), rheumatoid arthritis (RA), and Graves’ disease [59,60,61]. Notably, ICIs may amplify CD40L+ T cell expansion by disinhibiting T cell signaling, thereby hyperactivating CD40 pathways in target organs and recapitulating autoimmune-like pathology. Clinical-stage monoclonal antibodies targeting the CD40-CD40L axis have shown efficacy in autoimmune disorders [58], offering a precision strategy to suppress pathological immune responses while potentially mitigating irAEs.

Adenosine deaminase (ADA) has been recognized as a risk factor exclusively associated with high-grade irAEs, with robust co-localization evidence further establishing a causal relationship, thereby elucidating the specific mechanisms underlying the onset of high-grade irAEs. Adenosine, an immunosuppressive molecule, exerts its effects by binding to A2A and A2B receptors, which suppress T cell activation and promote Tregs function, consequently fostering an immunosuppressive tumor microenvironment (TME) that facilitates angiogenesis and metastasis [62]. ADA deaminates adenosine, converting it into inosine, thereby reducing adenosine concentrations in the TME and alleviating its inhibitory effects on immune cells [63]. Excessive immune cell infiltration is a key mechanism underlying irAEs. Notably, elevated ADA levels have been observed in patients with ICI-related hepatitis [64], thyroiditis [65], and encephalitis [66], indicating activation of the immune response. Consequently, ADA could serve as a promising biomarker for predicting high-grade irAEs, and its potential combination with immune checkpoint inhibitors may help balance the therapeutic efficacy against tumors with the risk of irAEs.

### 4.3. Growth Factors

Transforming growth factor β1 (TGFβ1) and TGFα both belong to the growth factor family, but they appear to have distinct roles in irAEs in our MR study. In line with Martinović et al. [67], TGFβ was a risk factor for irAEs, with significantly elevated baseline levels observed in melanoma patients with irAEs. TGFβ1 has been shown to promote tissue fibrosis and chronic inflammation, with the TGFβ/Smad signaling pathway being a key driver of pulmonary fibrosis in ICI-induced pneumonia [68,69]. In addition, in inflamed or immune-privileged sites, TGFβ enhances PD-L1-mediated generation of Tregs [70]. PD-L1 inhibitors may disrupt the balance between PD-L1 and TGFβ, potentially leading to immune-mediated damage to normal tissues.

TGFα exerts a protective effect against irAEs in our study, irrespective of their severity (high-grade or all-grade). As a ligand of the epidermal growth factor receptor (EGFR), TGFα is widely expressed in multiple cell types, including macrophages, epithelial cells, and mesenchymal stem cells (MSCs). By activating EGFR signaling, TGFα promotes epithelial proliferation and migration, thereby facilitating tissue repair [71]. Notably, MSC transplantation studies suggested its pivotal role in colitis recovery: TGFα expression was markedly upregulated in intestinal epithelial cells post-MSC intervention, accompanied by elevated TGFα mRNA levels in colonic tissues, indicating enhanced MSC paracrine activity that drives mucosal regeneration [72]. Furthermore, TGFα ameliorated myocardial ischemic injury, likely through modulating MSC paracrine functions and suppressing cardiomyocyte apoptosis [73]. In patients with acute lung injury/acute respiratory distress syndrome (ALI/ARDS), elevated TGFα levels in pulmonary edema fluid were correlated with their capacity to induce alveolar epithelial repair in vitro [74].

Although direct evidence linking TGFα to irAEs remains lacking, its biological roles in mucosal healing, anti-apoptosis, and stem cell regulation imply a potential protective effect against irAE-mediated tissue damage. Exogenously enhancing TGFα expression may thus represent a viable therapeutic strategy for mitigating irAEs, though further mechanistic and clinical validation is warranted.

Our study revealed no significant mediation of BMI’s effect on irAEs through plasma proteins, while preclinical evidence implicates alternative mechanistic pathways. In diet-induced obese murine models, upregulated PD-1 expression on T lymphocytes drives functional exhaustion, wherein subsequent anti-PD-1 therapy exacerbates psoriasiform dermatitis, directly establishing checkpoint dysregulation in obesity-dependent irAE pathogenesis [75]. Concurrently, adipose tissue remodeling fosters a chronic pro-inflammatory environment characterized by M1 macrophage polarization, CD8+ T cell infiltration, and Treg depletion, collectively causing systemic immune hyper-reactivity. This pre-existing inflammation may synergize with ICIs to potentiate autoimmunity. Pharmacokinetically, weight-based dosing risks supratherapeutic exposure in obese patients due to disproportionate adipose expansion relative to volume of distribution, potentially amplifying ICI toxicity [76,77]. Notably, clinical observations exhibit heterogeneity. A prospective cohort of nivolumab-treated Hodgkin lymphoma patients demonstrated no BMI-irAE association [78], whereas meta-analyses identify obesity as a risk factor for grade 3–4 irAEs [79]. This discrepancy likely stems from cohort-specific confounders (e.g., tumor immunogenicity, therapeutic regimens) or methodological limitations in covariate adjustment. Future investigations should delineate adipose distribution-dependent mechanisms of organ-specific irAEs and validate weight-adapted dosing or pathway-targeted anti-inflammatory interventions.

Our pathway analyses delineated distinct molecular landscapes between all-grade and high-grade irAEs. The enrichment of chemotaxis and granulocyte migration in all-grade irAEs underscored the pivotal role of innate immune cells (e.g., neutrophils) in initiating broad tissue inflammation [80]. In contrast, the specific enhancement of lymphocyte/monocyte chemotaxis in high-grade irAEs suggested dysregulated adaptive immunity, potentially driven by excessive T cell activation via immune receptor-ligand interactions. Notably, the IL-17 signaling pathway was exclusively enriched in high-grade irAEs, aligning with evidence that Th17-mediated inflammation underlies severe irAEs [8,81,82,83]. The concurrent enrichment of primary immunodeficiency pathways (e.g., CTLA4 variants) in high-grade irAEs implies that genetic predisposition may amplify immune dysregulation, where CTLA-4 pathway dysfunction drives widespread multi-organ lymphocyte infiltration, Treg cell deficiency, and autoantibody production [84,85].

Tissue-specific gene expression patterns (e.g., CSF1 in lung, TNFSF12 in ileum) may explain organ-specific irAE vulnerabilities. Our gene interaction network revealed potential associations between target plasma proteins (hub genes) and the pathogenesis of irAEs. Core genes were closely interconnected with peripheral genes through co-expression, genetic/physical interactions, and shared functional modules, suggesting that these genes may regulate immune cell activation and amplify inflammatory signaling to drive irAE pathology. Notably, the enrichment of “response to tumor necrosis factor” and “mononuclear cell proliferation” functions underscored the link between these networks and cytokine storm mechanisms [86]. TNFα cascade amplification and aberrant immune cell proliferation may exacerbate inflammatory dissemination and tissue damage via positive feedback loops [87]. This implied that irAE complexity arises not only from multi-gene interactions but also from dysregulated activation of inflammatory pathways, ultimately triggering severe clinical reactions in susceptible patients.

This study primarily relies on European population-derived GWAS and pQTL data for MR analysis of plasma proteins associated with irAEs, which may limit the generalizability of our findings. Importantly, emerging evidence from diverse ancestries reveals both convergent and divergent biomarker profiles. Consistent with our results, elevated baseline CD40L in melanoma patients with ICI-related dermatitis and increased IL-17 in pneumonia have been replicated in a US cohort, reinforcing the role of IL-17 signaling in irAE pathogenesis [88]. Parallel observations from Chinese lung cancer cohorts included enriched TNF-α pathway activity and higher plasma TNF levels [89], while elevated IL-10/CCL2 in elderly patients and ST6GAL1 in high-grade irAEs further highlight shared inflammatory axes [90]. Population-specific patterns are also evident, such as increased Angiopoietin-1 (Ang-1) and granulocyte colony-stimulating factor (G-CSF) in US studies [88], and markedly higher CD163—a macrophage activation marker linked to immune suppression—in Japanese melanoma patients with irAEs [91]. These findings collectively underscore the need to incorporate multi-ethnic cohorts in future research to refine predictive biomarkers across global populations and address ancestry-related heterogeneity in irAE mechanisms.

A notable strength of this study is the integrative use of MVMR, colocalization, and pathway analyses. This multi-modal approach generated robust genetic evidence to identify BMI-independent plasma protein biomarkers (e.g., CXCL9, TNFSF12, CCL25) and mechanistically delineate their contributions to irAE pathogenesis. However, several limitations still existed. First, the reliance on European-centric GWAS and pQTL data limits generalizability to non-European populations, where genetic and environmental heterogeneity may alter protein-irAE associations. Second, residual confounding from unmeasured immune mediators or cell-type-specific protein modifications could bias causal estimates, as circulating protein levels may incompletely reflect tissue-specific dynamics. Finally, the risk or protective role of proteins lacks direct experimental validation. Preclinical models and longitudinal human studies are needed to confirm its mechanistic contribution to irAE mitigation.

## 5. Conclusions

A notable strength of this study is the integrative use of MVMR, colocalization, and pathway analyses. This multi-modal approach generated robust genetic evidence to identify BMI-independent plasma protein biomarkers (e.g., CXCL9, TNFSF12, CCL25) and mechanistically delineate their contributions to irAE pathogenesis. However, several limitations still existed. First, the reliance on European-centric GWAS and pQTL data limits generalizability to non-European populations, where genetic and environmental heterogeneity may alter protein–irAE associations. Second, residual confounding from unmeasured immune mediators or cell-type-specific protein modifications could bias causal estimates, as circulating protein levels may incompletely reflect tissue-specific dynamics. Furthermore, due to constraints of public data sources, the absence of paired individual-level protein-outcome data impedes clinical validation of predictive models (e.g., ROC analysis). Finally, while our MR analysis supports causal protein-irAE relationships, experimental validation of their specific mechanisms remains essential. Future studies should prioritize in vitro and in vivo models to elucidate the direct modulation of immune pathways in irAEs.

## Figures and Tables

**Table 1:** Bayesian co-localization analysis on seven potential causal proteins in all-grade irAEs.

Protein	SNP	Co-Localization (coloc.abf)				
		PP.H0.abf	PP.H1.abf	PP.H2.abf	PP.H3.abf	SNP.PP.H4
CCL20	rs10207134	1.03 × 10^−9^	0.704	3.58 × 10^−10^	0.244	0.052
CD40	rs1883832	3.29 × 10^−273^	0.252	1.16 × 10^−273^	0.033	**0.716**
CSF1	rs17610659	6.14 × 10^−40^	0.718	1.74 × 10^−40^	0.204	0.079
CXCL9	rs4241577	9.49 × 10^−23^	0.710	3.04 × 10^−23^	0.227	0.063
TGFB1	rs73045269	6.48 × 10^−30^	0.764	1.58 × 10^−30^	0.187	0.050
TGFA	rs72912115	9.81 × 10^−9^	0.735	2.68 × 10^−9^	0.200	0.065
TNFSF12	rs62061198	3.55 × 10^−23^	0.049	3.80 × 10^−24^	0.092	**0.859**

**Table 2:** Bayesian co-localization analysis on five potential causal proteins in high-grade irAEs.

Protein	SNP	Co-Localization (coloc.abf)				
		PP.H0.abf	PP.H1.abf	PP.H2.abf	PP.H3.abf	SNP.PP.H4
ADA	rs11555566	3.77 × 10^−10^	0.045	0.105	0.034	**0.817**
CCL20	rs10207134	1.00 × 10^−9^	0.684	3.84 × 10^−10^	0.262	0.054
CCL25	rs2032887	2.62 × 10^−11^	0.002	0.132	0.127	**0.739**
CXCL10	rs71607345	1.50 × 10^−12^	0.729	4.55 × 10^−13^	0.221	0.050
TGFA	rs72912115	8.99 × 10^−9^	0.673	3.10 × 10^−9^	0.232	0.095

## Data Availability

All the data used in this study were publicly available. No datasets were generated or analyzed during the current study. Data from GWAS are accessible through the following reported sources: the deCODE GENETICS Consortium (https://www.decode.com/summarydata/ accessed on 2 June 2024), Full per-protein GWAS summary statistics are available for download at the EBI GWAS Catalog (accession numbers GCST90274758 to GCST90274848). MR GWAS summary statistics for BMI were downloaded from OpenGWAS (https://gwas.mrcieu.ac.uk/datasets accessed on 2 June 2024).

## Supplementary Materials

The following supporting information can be downloaded at: https://www.mdpi.com/article/10.3390/biomedicines13071717/s1. Table S1. Eligible instrumental variables (IVs) associated with plasma proteins; Table S2. Positive SNPs in plasma proteins-all-grade irAEs MR analysis; Table S3. Positive results of primary MR analysis between plasma proteins and all-grade irAEs; Table S4. Heterogeneity analysis between plasma protein and all-grade irAEs; Table S5. Pleiotropy analysis between plasma protein and all-grade irAEs; Table S6. Positive SNPs in plasma proteins-high-grade irAEs MR analysis; Table S7. Positive results of primary MR analysis between plasma proteins and high-grade irAEs; Table S8. Heterogeneity analysis between plasma protein and high-grade irAEs; Table S9. Pleiotropy analysis between plasma protein and high-grade irAEs; Table S10. Results of reverse MR analysis between all-grade irAEs and the identified significant plasma proteins; Table S11. Results of reverse MR analysis between high-grade irAEs and the identified significant plasma proteins; Table S12 MVMR combined BMI and plasma proteins for all-grade irAEs; Table S13. MVMR combined BMI and plasma proteins for high-grade irAEs; Table S14. Two-step network MR using plasma proteins, BMI and all-grade irAEs; Table S15. Two-step network MR using plasma proteins, BMI and high-grade irAEs; Table S16. Results of gene set enrichment analysis (GTEx v8) using FUMA for proteins for all-grade irAEs; Table S17. Results of gene set enrichment analysis (GTEx v8) using FUMA for proteins for high-grade irAEs; Table S18. Results for 8 prioritized proteins on all-grade irAEs using GeneMANIA for physical interaction (1), function (2), and involving pathway (3); Table S19. Results for 5 prioritized proteins on high-grade irAEs using GeneMANIA for physical interaction (1), function (2), and involving pathway (3); Table S20. Causal weights of positive proteins for risk prediction in all-grade irAEs and high-grade irAEs.
